# Microbial Distribution and Antibiotic Susceptibility of Lower Respiratory Tract Infections Patients From Pediatric Ward, Adult Respiratory Ward, and Respiratory Intensive Care Unit

**DOI:** 10.3389/fmicb.2020.01480

**Published:** 2020-06-30

**Authors:** Nan Duan, Jialin Du, Chenwei Huang, Haixia Li

**Affiliations:** Department of Clinical Laboratory, Peking University First Hospital, Beijing, China

**Keywords:** lower respiratory tract infections, antibiotic susceptibility, *Pseudomonas aeruginosa*, *Staphylococcus aureus*, *Klebsiella pneumoniae*

## Abstract

**Introduction:**

Lower respiratory tract infections (LRTIs) account for significant morbidity and mortality in patients admitted to hospitals worldwide, especially in children and elderly. The prevalent microorganisms and antibiotic susceptibility were investigated among LRTI patients from the pediatric ward, adult respiratory ward, and respiratory intensive care unit (RICU) in order to achieve more efficient treatment protocols and better recovery.

**Methods:**

In this retrospective cross-sectional study (January 2016 to December 2019), 4,161 positive culture samples out of 18,798 different specimens (9,645 respiratory tract samples and 9,153 blood samples) from LRTI patients were analyzed for pathogen incidence and antibiotic sensitivity.

**Results:**

Among the respiratory tract cultures, the frequency of Gram-negative bacterial strains was higher than Gram-positive bacterial strains. *Pseudomonas aeruginosa* was the dominant pathogen in both the adult respiratory ward (*n* = 156, 21.49%) and RICU (*n* = 975, 35.67%), whereas *Staphylococcus aureus* (*n* = 66, 19.19%) was the most common bacterium in the pediatric ward. Among the blood cultures, Gram-positive bacteria remained the major microorganisms involved in LRTIs, and the most frequent pathogen was *Staphylococcus epidermidis* (*n* = 59, 47.20%) in the pediatric ward and *Staphylococcus aureus* (*n* = 10, 21.8%) in adult respiratory ward. However, Gram-negative bacteria were the main pathogens in the RICU, of which *Klebsiella pneumoniae* (*n* = 51, 27.57%) is the most prevalent. *Pseudomonas aeruginosa* of LRTI patients remained highly susceptible (>70%) to routine antibiotics in pediatric ward. However, it only had high susceptibility to amikacin, tobramycin, gentamicin in both the adult respiratory ward and RICU and its antibiotic sensitivity to meropenem and imipenem was moderate in the adult respiratory ward and mild (<30%) in the RICU. *Staphylococcus aureus* isolated from LRTI patients was highly susceptible to linezolid, daptomycin, teicoplanin, vancomycin, tigecycline, rifampicin, and trimethoprim/sulfamethoxazole in all three wards, moderately susceptible to gentamicin in both the adult respiratory ward and RICU and to clindamycin, oxacillin, moxifloxacin only in the adult respiratory ward.

**Conclusions:**

Microbial distribution and their patterns of antibiotic susceptibility revealed a high divergence among LRTI patients admitted to different wards in this hospital. Thus, different antibiotic therapies should be considered for distinct age groups.

## Introduction

Lower respiratory tract infections (LRTIs) are common among patients worldwide, and the most major cause of pneumonia and bronchiolitis in hospitalization. There are 3.5 million deaths attributed to and 79 million disability-adjusted life-years (DALYs) lost to LRTIs which are associated with high overall morbidity and mortality in adults, especially in patients older than 70 years old (Troeger et al., 2017; Mahendra et al., 2018). The elderly have an increased risk of developing LRTIs compared to young adults. Furthermore, it is also reported that children admitted with LRTIs had more severe respiratory disease and a longer recovery period (Trenholme et al., 2013), even a quarter of them suffering respiratory sequelae (Nathan et al., 2020). Antibiotics are commonly used to treat LRTIs, but clinical management of LRTIs is difficult due to antibiotic resistance.

Currently, irrational administration of antimicrobial agents and inappropriate antibiotics therapy introduced serious antibiotic resistance, increased hospital mortality rate, and healthcare-related expenditure for LRTI patients (Harris et al., 2016). Chinese researchers revealed that of the outpatients prescribed antibiotics, 74.0% were prescribed one antibiotic, 23.3% were prescribed two antibiotics and 2.0% were prescribed three or more antibiotics (Yin et al., 2013). Thus the effective selection and use of antibiotics newly has become a huge challenge for all physicians. Thoroughly understanding prevalent microorganisms and their susceptibility patterns will improve the management of patients with LRTIs worldwide.

Antibiotics can also be performed in surveillance programs to improve treatment protocols and indicate the trends in microbial resistance (Beam et al., 2014). However, a comparison of relative frequency and microbiological characteristics of the pathogens in pediatric versus adult patients with LRTIs admitted to respiratory wards and respiratory intensive care units (RICU) had not been previously investigated in China. The purpose of this study was to investigate the prevalent microorganisms and patterns of antimicrobial sensitivity among LRTI patients from the pediatric ward, adult respiratory ward and RICU to achieve more efficient treatment protocols and better recovery in our hospital.

## Materials and Methods

### Subjects

We conducted a retrospective cross-sectional study in Department of Pediatrics, Department of Respiratory Medicine and Department of RICU in Peking University First Hospital (a large-scale tertiary general hospital) from 1 January, 2016 to 31 December, 2019. A total of 4,161 positive culture samples from 18,841 different specimens (9,645 respiratory tract samples and 9,196 blood samples) were obtained from patients diagnosed with LRTIs in pediatric wards, adult respiratory wards, and RICU (Table 1). The respiratory tract samples (sputum or bronchoalveolar lavage fluid, BALF) and blood samples were collected before the start of antimicrobial treatment. The diagnoses were based on the diagnostic criteria of bronchiolitis, community-acquired pneumonia (CAP) and hospital-acquired pneumonia (HAP) by the WHO definition. This study has been reviewed and approved by Institutional Ethics Committee of Peking University First Hospital, and a review in terms of patient consent was not needed because of the retrospective observational design according to the committee decision.

**TABLE 1:** LRTI patient characteristics, respiratory tract, and blood cultures of different wards.

	**Pediatric ward**	**Adult respiratory ward**	**RICU**
Patients (*n*)	1,737	1,655	506
Male (%)	55.90	63.20	62.06
Age (years)	6.44 ± 2.15	52.20 ± 11.52	74.04 ± 16.18
Current symptoms (%)			
Chest discomfort	6.51	18.97	27.08
Cough	52.96	72.08	78.06
Dyspnea	29.02	80.97	98.22
Respiratory tract cultures			
Positive/total isolates (*n*/*N*)	344/2,195	726/3,151	2,734/4,299
Detection rate (%)	15.67	23.04	63.59
Blood cultures			
Positive/total isolates (*n*/*N*)	125/5,926	47/1,253	185/1,974
Detection rate (%)	2.11	3.75	9.37
Resistance pattern (*n*/%)			
Positive cultures (*n*)	469	773	2,919
ESBL	22/4.69	10/1.29	59/2.02
CRE	5/1.07	9/1.16	358/12.26
CRAB	4/0.85	54/6.99	573/19.63
MRSA	18/3.84	17/2.20	44/1.51
MRCNS	55/11.73	11/1.42	50/1.71
PRSP	8/1.71	2/0.26	0/0

*Continuous values are expressed as mean ± SD. RICU, respiratory intensive care unit; ESBL, extended-spectrum β-lactamase; CRE, carbapenem-resistant *Enterobacteriaceae*; CRAB, carbapenem-resistant *Acinetobacter baumannii*; MRSA, methicillin-resistant *Staphylococcus aureus*; MRCNS, methicillin-resistant coagulase-negative *staphylococci*; PRSP, penicillin-resistant *Streptococcus pneumoniae*.*

### Microorganism Identification and Drug-Susceptibility Test

Microorganism cultures were conducted according to the routine standard operation procedure (SOP) by the Department of Clinical Laboratory in our hospital. Each respiratory tract specimen was inoculated on a blood agar media (Columbia A), a China blue lactose rosolic acid agar plate and a chocolate agar plate (with Vancomycin) (OXOID, Thermo Fisher Scientific) using the streak plate technique and incubated under a CO_2_-enriched atmosphere for 24–48 h at 37°C. Blood specimen was inoculated into a commercial culture bottle and analyzed by an automated monitoring system for bacterial detection (BD BACTEC^TM^, BD, United States). The incubation was continued until a positive result was observed or up to a maximum of 5 days.

Positive bacterial cultures from patients’ respiratory tract and blood samples collected within the first 24 h of admission were tested for type of pathogens and sensitivity to antibiotics. The microorganisms were identified to species level by standard biochemical methods including matrix-assisted laser desorption ionization-time of flight mass spectrometry (MALDI-TOF MS) method (Bruker Daltonics, United States) and automated methods with the VITEK system (BioMérieux, France). The bacterial isolates were tested for antimicrobial susceptibility performed by VITEK 2 Compact (BioMérieux, France) or the manual Kirby–Bauer (K–B) disk diffusion method. The selection of VITEK susceptibility card types (BioMérieux, France) was as follows: AST-P639 for Gram-Positive bacteria, AST-GN09 for *Pseudomonas aeruginosa*, and AST-N335 for other Gram-negative bacteria according to the needs of clinicians. Interpretation of drug susceptibility results were based on the Clinical Laboratory Standards Institute (CLSI, United States) standard.

### Statistic Analysis

Continuous values are expressed as mean ± SD. Microbial distribution and antibiotic susceptibility data were presented as percentages. The Chi-square (χ^2^) or Fisher exact tests were performed to compare differences between groups using SPSS version 16.0 software (SPSS Inc., Chicago, IL, United States). A *P* < 0.05 was considered statistically significant.

## Results

### Respiratory Tract and Blood Cultures From LRTI Patients

From 1 January, 2016 to 31 December, 2019, the frequencies of reportedly positive cultures of LRTI patients were 344 (detection rate: 15.67%) out of 2,195 respiratory tract cultures and 125 (2.11%) out of 5,926 blood cultures in the pediatric ward; 726 (23.04%) out of 3,151 respiratory tract cultures and 47 (3.75%) out of 1,253 blood cultures in the adult respiratory ward; and 2,734 (63.59%) out of 4,299 respiratory tract cultures and 185 (9.37%) out of 1,974 blood cultures in the RICU. The prevalence of positive cultures of LRTI patients for different wards was summarized in Table 1. RICU cultures had the highest detection rates (both in respiratory tract and blood cultures), significantly higher than that in pediatric ward and adult respiratory ward (all *P* < 0.05 each). The most prevalent resistance patterns were methicillin-resistant coagulase-negative *staphylococci* (MRCNS) (11.73%) in the pediatric ward, carbapenem-resistant *Acinetobacter baumannii* (CRAB) (6.99%) in the adult respiratory ward, and CRAB (19.63%) and carbapenem-resistant *Enterobacteriaceae* (CRE) (12.26%) in the RICU.

**FIGURE 1:**
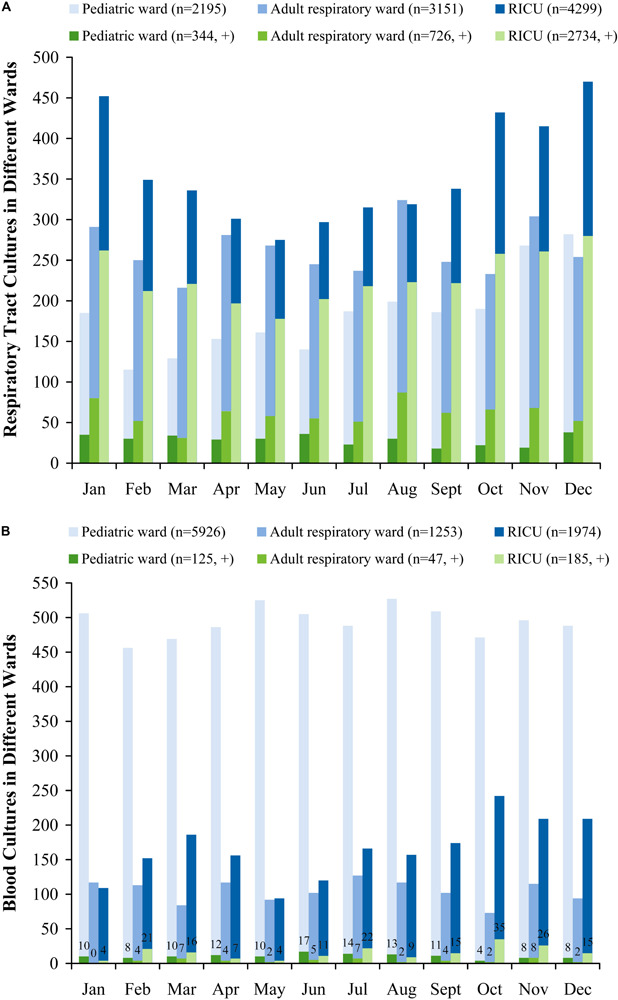
Monthly distribution of sampling **(A)** respiratory tract cultures, **(B)** blood cultures in patients with lower respiratory tract infections from the pediatric ward, adult respiratory ward, and respiratory intensive care unit (RICU). More respiratory tract and blood cultures of LRTI patients from three wards were performed in all seasons (January to December) than in cold seasons (October to December). RICU, respiratory intensive care unit.

More respiratory tract and blood specimens of LRTI patients from three wards were performed in all seasons (January to December) than in cold seasons (October to December), as evidenced by 804 (positive, 317) of monthly mean respiratory tract cultures and 763 (positive, 30) of blood cultures in all seasons, and 949 (positive, 355) and 799 (positive, 36) in cold seasons, respectively (Figure 1).

### Distribution of Pathogens in the Pediatric Ward

**FIGURE 2:**
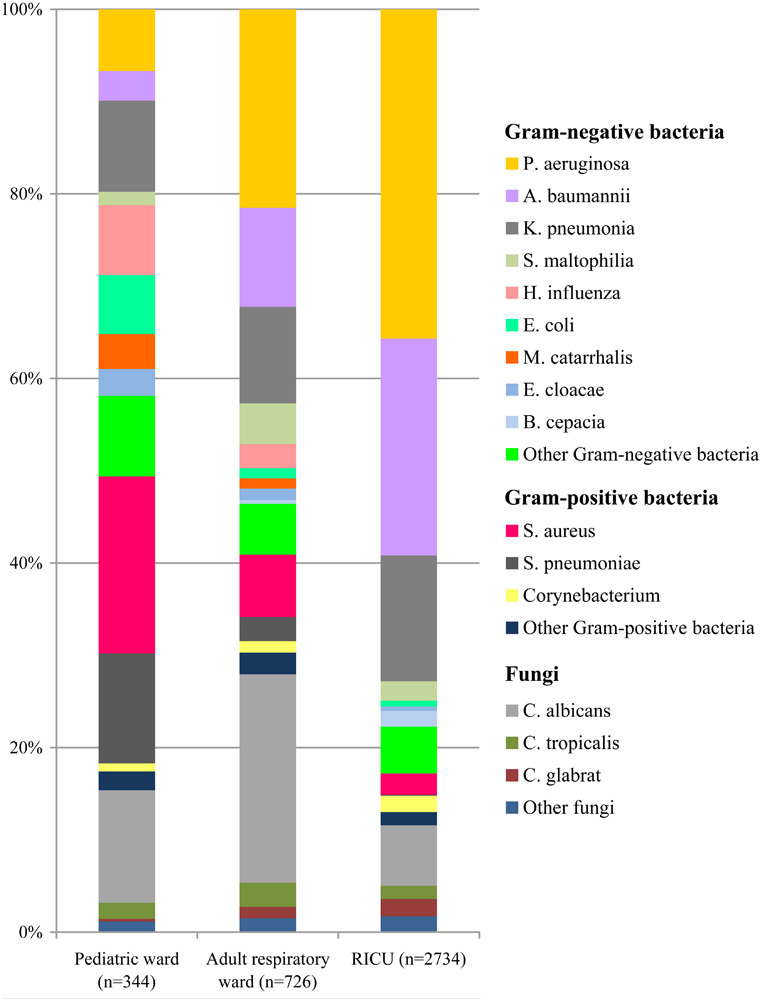
Distribution of microorganisms in positive respiratory tract cultures of lower respiratory tract infection patients from the pediatric ward, adult respiratory ward, and respiratory intensive care unit (RICU). Gram-negative bacteria remained the major pathogens among the respiratory tract cultures. *P. aeruginosa*, *Pseudomonas aeruginosa*; *A. baumannii*, *Acinetobacter baumannii*; *K. pneumonia*, *Klebsiella pneumonia*; *S. maltophilia*, *Stenotrophomonas maltophilia*; *H. influenza*, *Haemophilus influenza*; *E. coli*, *Escherichia coli*; *M. catarrhalis*, *Moraxella catarrhalis*; *E. cloacae*, *Enterobacter cloacae*; *B. cepacia*, *Burkholderia cepacia*; *S. aureus*, *Staphylococcus aureus*; *S. pneumonia*, *Streptococcus pneumonia*; *C. albicans*, *Candida albicans*; *C. tropicalis*, *Candida tropicalis*; *C. glabrata*, *Candida glabrata.*

The profile of the pathogens isolated from LRTI patients’ respiratory tract specimens was summarized in Figure 2. In the pediatric ward, the most five frequent bacterial strains of respiratory tract were *Staphylococcus aureus* (*n* = 66, 19.19%), *Streptococcus pneumoniae* (*n* = 41, 11.92%), *Klebsiella pneumoniae* (*n* = 34, 9.89%), *Pseudomonas aeruginosa* (*n* = 23, 6.69%), and *Haemophilus influenzae* (*n* = 26, 7.56%). Other pathogens included *Escherichia coli* at 6.40%, *Moraxella catarrhalis* at 3.78%, and *Enterobacter cloacae* at 2.91%.

**FIGURE 3:**
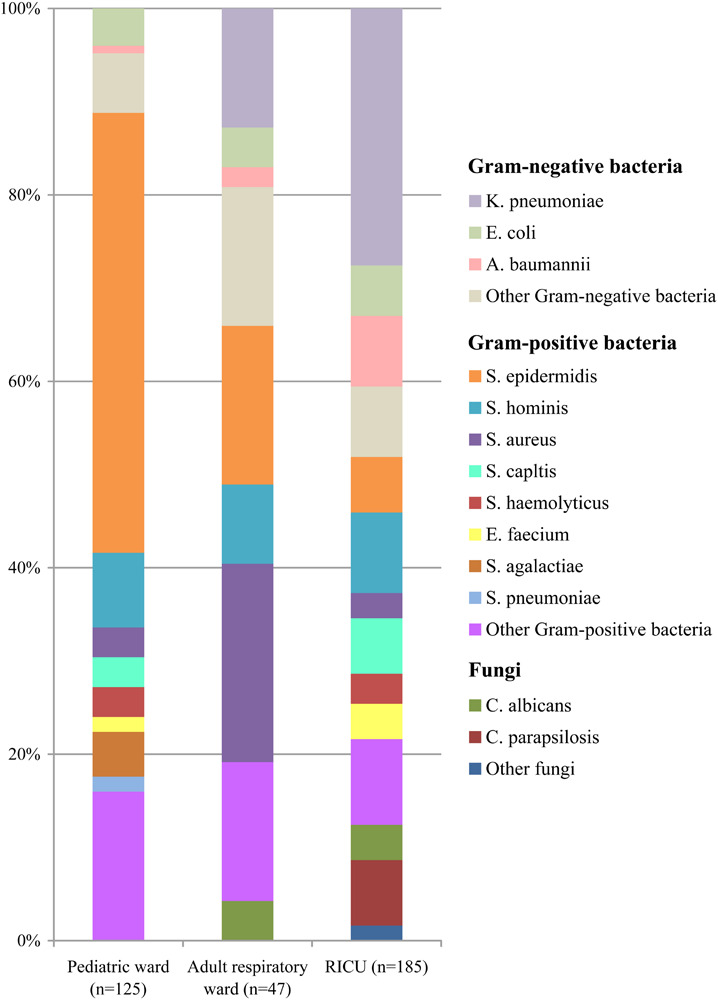
Distribution of microorganisms in positive blood cultures of lower respiratory tract infection patients from the pediatric ward, adult respiratory ward, and respiratory intensive care unit (RICU). Among the blood cultures, the frequency of Gram-positive bacteria was higher than Gram-negative bacteria in the pediatric ward and adult respiratory ward, and Gram-negative bacterial strains remained the major pathogens in the RICU. *K. pneumonia*, *Klebsiella pneumonia*; *E. coli*, *Escherichia coli*; *A. baumannii*, *Acinetobacter baumannii*; *S. epidermidis*, *Staphylococcus epidermidis*; *S. hominis*, *Staphylococcus hominis*; *S. aureus*, *Staphylococcus aureus*; *S. capltis*, *Staphylococcus capltis*; *S. haemolyticus*, *Staphylococcus haemolyticus*; *E. faecium*, *Enterococcus faecium*; *S. agalactiae*, *Streptococcus agalactiae*; *S. pneumonia*, *Streptococcus pneumoniae*; *C. albicans*, *Candida albicans*; *C. parapsilosis*, *Candida parapsilosis.*

The types and numbers of bacterial strains isolated from blood cultures were shown in Figure 3. Gram-positive bacteria (*n* = 111, 88.80%) accounted for more than 80% in blood specimens of patients admitted to the pediatric ward, and *Staphylococcus epidermidis* (*n* = 59, 47.20%) was the most common pathogen. *Staphylococcus hominis* (*n* = 10, 8.00%) was the second most common bacterium, followed by *Streptococcus agalactiae* (*n* = 6, 4.80%) and *E. coli* (*n* = 5, 4.00%).

### Distribution of Pathogens in the Adult Respiratory Ward

Among the respiratory tract specimens of LRTI patients (Figure 2) admitted to the adult respiratory ward, *P. aeruginosa* (*n* = 156, 21.49%) was the most common pathogen. *Acinetobacter baumannii* (*n* = 78, 10.74%) was the second most common bacterium, followed by *K. pneumoniae* (*n* = 76, 10.46%), *S. aureus* (*n* = 49, 6.75%), and *Stenotrophomonas maltophilia* (*n* = 32, 4.41%).

The profiles of the microorganisms isolated from blood cultures were shown in Figure 3. The four most frequent microorganisms were *S. aureus* (*n* = 10, 21.8%), *S. epidermidis* (*n* = 8, 17.02%), *K. pneumoniae* (*n* = 6, 12.77%), and *S. hominis* (*n* = 4, 8.51%) in the adult respiratory ward.

### Distribution of Pathogens in the RICU

The types and numbers of bacterial strains of the RICU patients’ the respiratory tract cultures were shown in Figure 2. Gram-negative bacteria (*n* = 2,264) accounted for 82.81% of isolations, and the four most predominant pathogens were *P. aeruginosa* (*n* = 975, 35.67%), *A. baumannii* (*n* = 643, 23.51%), *K. pneumoniae* (*n* = 373, 13.64%), and *S. aureus* (*n* = 63, 2.30%).

In the RICU blood cultures (Figure 3), the dominant pathogen was *K. pneumoniae* (*n* = 51, 27.57%), followed by *S. hominis* (*n* = 16, 8.65%), *A. baumannii* (*n* = 14, 7.57%), *S. epidermidis* (*n* = 11, 5.95%), and *Staphylococcus capltis* (*n* = 11, 5.95%).

### Comparison of Microorganisms Involved in LRTI Patients

Among the respiratory tract cultures (Figure 2), Gram-negative bacteria remained the major microorganisms involved in LRTIs, and their frequency was higher than Gram-positive bacteria. The total proportion of Gram-negative bacteria isolated from LRTI patients was significantly higher in the RICU (82.81%) than in the pediatric ward (50.58%) (*P* < 0.05) and adult respiratory ward (59.1%) (*P* < 0.05). *P. aeruginosa* was the dominant pathogen in both the adult respiratory ward and RICU, whereas *S. aureus* was the most common bacterium in the pediatric ward. Fungal pathogens in the pediatric ward, adult respiratory ward and RICU were respectively at 15.41%, 27.96% and 11.59%, and *Candida albicans* was the most prevalent in all three wards.

Among the blood cultures (Figure 3), the frequency of Gram-positive bacterial strains (especially *S. epidermidis*) was higher than Gram-negative bacterial strains in both the pediatric ward and adult respiratory ward, and the proportion of Gram-positive bacteria isolated from LRTI patients was significantly higher in the pediatric ward than in the adult respiratory ward and RICU (*P* < 0.05). However, Gram-negative bacteria remained the major pathogens involved in LRTIs from the RICU, accounting for 48.11% (*n* = 89). Fungal pathogens in the pediatric ward, adult respiratory ward and RICU were at 0%, 4.26%, and 12.43%, respectively. *Candida albicans* and *Candida parapsilosis* were respectively the most prevalent.

### Antibiotic Susceptibility of Major Bacterial

**FIGURE 4:**
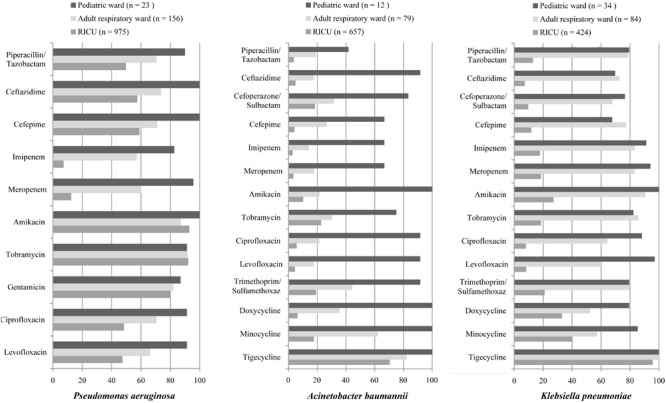
Major Gram-negative bacteria isolated and their drug-susceptibility rates in patients with lower respiratory tract infections from the pediatric ward, adult respiratory ward, and respiratory intensive care unit (RICU). The antimicrobial susceptibility performed by VITEK 2 Compact or the manual Kirby–Bauer (K–B) disk diffusion method. RICU, respiratory intensive care unit.

Drug-susceptibility of Gram-negative *P. aeruginosa*, *A. baumannii*, and *K. pneumoniae* was selected as shown in Table 2 and Figure 4. *P. aeruginosa* of LRTI patients remained highly susceptible (>70%) to all tested antibiotics in pediatric ward. However, only high susceptibility to amikacin, tobramycin, gentamicin, and moderate susceptibility to piperacillin/tazobactam, cefepime, ceftazidime, levofloxacin, ciprofloxacin were observed in both the adult respiratory ward and RICU. Moreover, its antibiotic sensitivity to meropenem and imipenem was moderate in the adult respiratory ward, and mild (<30%) in the RICU. *A. baumannii* remained highly or moderately susceptible to tested antibiotics in the pediatric ward, whereas it exhibited relatively low drug-susceptibility to antibiotics except tigecycline in the respiratory ward and RICU. *K. pneumoniae* was highly susceptible to tigecycline of patients hospitalized with LRTIs in all three wards. Its drug-susceptibility to other antibiotics, such as piperacillin/tazobactam imipenem, meropenem, amikacin, tobramycin and trimethoprim/sulfamethoxaz was relatively high in both the pediatric ward and adult respiratory ward, but mildly susceptible to routine antibiotics in the RICU.

**TABLE 2:** Drug-susceptibility of major Gram-negative bacteria of LRTI patients from different wards (%).

**Antibacterials**	***Pseudomonas aeruginosa***			***Acinetobacter baumannii***			***Klebsiella pneumoniae***		
	**PW (*n* = 23)**	**ARD (*n* = 156)**	**RICU (*n* = 975)**	**PW (*n* = 12)**	**ARD (*n* = 79)**	**RICU (*n* = 657)**	**PW (*n* = 34)**	**ARD (*n* = 84)**	**RICU (*n* = 424)**
Piperacillin/Tazobactam	89.96	70.51	49.64	41.67	18.99	3.65	79.41	78.57	12.97
Ceftazidime	100.00	73.72	57.44	91.67	17.72	4.87	69.70	72.62	7.31
Cefoperazone/Sulbactam	–	–	–	83.33	31.65	18.41	76.47	67.85	9.67
Cefepime	100.00	71.15	58.94	66.67	26.58	4.11	67.65	77.38	11.79
Imipenem	82.61	57.05	7.28	66.67	13.97	2.73	91.18	83.33	17.69
Meropenem	95.65	60.26	12.41	66.67	17.72	3.35	94.11	83.33	18.40
Amikacin	100.00	87.18	92.92	100.00	21.52	10.20	100	90.48	27.12
Tobramycin	91.30	91.67	92.13	75.00	30.38	22.68	82.35	85.71	18.40
Gentamicin	86.96	82.05	80.10	–	–	–	–	–	
Ciprofloxacin	91.30	70.51	48.41	91.67	21.52	5.63	88.24	64.29	8.02
Levofloxacin	91.30	66.26	47.38	91.67	17.72	4.41	97.06	60.71	8.25
Trimethoprim/Sulfamethoxaz	–	–	–	91.67	44.30	19.03	79.41	79.76	20.99
Doxycycline	–	–	–	100.00	35.44	6.24	79.41	52.38	33.02
Minocycline	–	–	–	100.00	62.03	17.66	85.29	57.14	40.09
Tigecycline	–	–	–	100.00	82.28	70.47	100.00	100.00	95.75

*PW, pediatric ward; ARD, adult respiratory ward; RICU, respiratory intensive care unit.*

**FIGURE 5:**
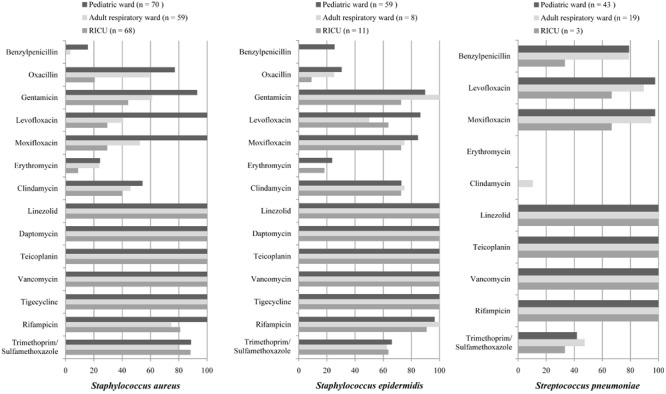
Major Gram-positive bacteria isolated and their drug-susceptibility rates in patients with lower respiratory tract infections from the pediatric ward, adult respiratory ward, and respiratory intensive care unit (RICU). The antimicrobial susceptibility performed by VITEK 2 Compact or the manual Kirby–Bauer (K–B) disk diffusion method. RICU, respiratory intensive care unit.

The antibiotic sensitivity of Gram-positive *S. aureus, S. epidermidis, and S. pneumoniae* was selected as shown in Table 3 and Figure 5. As a predominant Gram-positive bacterium, *S. aureus* isolated from LRTI patients was highly susceptible (>70%) to linezolid, daptomycin, teicoplanin, vancomycin, tigecycline, rifampicin, and trimethoprim/sulfamethoxazole in all three wards, moderately susceptible to gentamicin in the adult respiratory ward and RICU and to clindamycin, oxacillin, moxifloxacin in the adult respiratory ward. However, its sensitivity to other antibiotics was relatively low in the RICU. *S. epidermidis* was one of the main microorganisms in positive blood cultures. It exhibited high drug-susceptibility to gentamicin, moxifloxacin, clindamycin, linezolid, daptomycin, teicoplanin, vancomycin, tigecycline, and rifampicin, moderate sensitivity to trimethoprim/sulfamethoxazole, and mild sensitivity to benzylpenicillin, oxacillin, and erythromycin among patients with LRTIs in the pediatric ward, adult respiratory ward, and RICU. *S. pneumoniae* was the second most common bacterium in the pediatric ward, and its sensitivity to routine antibiotics except trimethoprim/sulfamethoxazole was high in the pediatric ward and respiratory ward.

**TABLE 3:** Drug-susceptibility of major Gram-positive bacteria of LRTI patients from different wards (%).

**Antibacterials**	***Staphylococcus aureus***			***Staphylococcus epidermidis***			***Streptococcus pneumoniae***		
	**PW (*n* = 70)**	**ARD (*n* = 59)**	**RICU (*n* = 68)**	**PW (*n* = 59)**	**ARD (*n* = 8)**	**RICU (*n* = 11)**	**PW (*n* = 43)**	**ARD (*n* = 19)**	**RICU (*n* = 3)**
Benzylpenicillin	15.71	3.39	0.00	25.42	0.00	0.00	79.07	78.95	33.33
Oxacillin	77.14	59.32	20.59	30.51	25.00	9.09	–	–	
Gentamicin	92.86	61.02	44.12	89.83	100.00	72.73	–	–	–
Levofloxacin	100.00	40.68	29.41	86.44	50.00	63.63	97.67	89.47	66.67
Moxifloxacin	100.00	52.54	29.41	84.75	75.00	72.73	97.67	94.74	66.67
Erythromycin	24.29	23.72	8.82	23.73	0.00	18.18	0.00	0.00	0.00
Clindamycin	54.29	45.76	39.71	72.89	75.00	72.73	0.00	10.53	0.00
Linezolid	100.00	100.00	100.00	100.00	100.00	100.00	100.00	100.00	100.00
Daptomycin	100.00	100.00	100.00	100.00	100.00	100.00	–	–	–
Teicoplanin	100.00	100.00	100.00	100.00	100.00	100.00	100.00	100.00	100.00
Vancomycin	100.00	100.00	100.00	100.00	100.00	100.00	100.00	100.00	100.00
Tigecycline	100.00	100.00	100.00	100.00	100.00	100.00	–	–	–
Rifampicin	100.00	74.57	80.88	96.61	100.00	90.90	100.00	100.00	100.00
Trimethoprim/Sulfamethoxazole	88.57	79.66	88.24	66.10	62.50	63.64	41.86	47.37	33.33

*PW, pediatric ward; ARD, adult respiratory ward; RICU, respiratory intensive care unit.*

## Discussion

Respiratory infections account for significant morbidity, mortality, and healthcare-related expenditure in patients admitted to hospitals worldwide. LRTIs are common bacterial infections leading to large antibiotic use and hospitalization, which account for 3 to 5% of deaths in adults, especially in patients over the age of 60 years (Xia et al., 2012). It is reported LRTIs such as CAP is one of the most frequent infectious diseases in children and the leading cause of death in less than 5 years old (Yun et al., 2019). Microbiological cultures of respiratory tract and blood specimens provided clinically relevant information concerning the identity and analysis of microorganisms with their susceptibility to antibiotics. With thoroughly microbiological analysis, we observed that the distribution and drug susceptibility of LRTI pathogens exhibited a high divergence among the pediatric ward, adult respiratory ward and RICU in our hospital. Thus, different antibiotic therapies for distinct age groups were suggested, and therapy guidelines should match pathogen distribution and antibiotic sensitivity.

In this study, we conducted a retrospective cross-sectional study for 4 years in Department of Pediatrics, Department of Respiratory Medicine and Department of RICU to analyze the positive respiratory tract and blood cultures isolated from LRTI patients. RICU cultures had the highest positive frequency (63.59% in respiratory tract specimens and 9.37% in blood specimens), similar to the findings in an Indian hospital where the isolation rates were different for different biologic specimens: sputum culture (30.92%), BALF (75%), and blood culture (12.5%) (Mahendra et al., 2018). Furthermore, the detection rate of RICU was significantly higher than that in pediatric ward and adult respiratory ward (*P* < 0.05), consistent with a previous report indicating that the elderly have an increased risk of developing LRTIs compared to young adults and nosocomial infections are very prevalent in patients admitted to ICU (Wałaszek et al., 2018).

In the present study, the profile of the pathogens from respiratory tract specimens showed that Gram-negative bacteria remained the major microorganisms involved in LRTIs, and the frequency of Gram-negative bacteria was higher than Gram-positive bacteria. *S. aureus* and *S. pneumoniae* were the predominant bacteria, followed by *K. pneumoniae* and *H. influenzae* in our pediatric ward. Similarly in Africa and Asia, it is revealed that the commonest bacterial pathogens of children hospitalized with LRTIs were *S. aureus*, *H. influenza*, and *S. pneumoniae* (Marangu and Zar, 2019; Pneumonia Etiology Research for Child Health (Perch) Study Group, 2019), as other bacterial pathogens reported from low-and-middle-income countries’ settings included *K. pneumoniae* and *E. coli* (Harris et al., 2019). The Global Burden of Disease (GBD) data showed 64.1% of LRTI deaths in children younger than 5 years were attributed to bacterial etiology, specifically *S. pneumoniae* and *H. influenzae* (GBD 2015 Lower Respiratory Infections Collaborators, 2017). In the adult respiratory ward and RICU of our hospital, Gram-negative bacteria remained the main microorganisms of LRTIs, while the total infection rate was significantly higher in the RICU (82.81%) than in the adult respiratory ward (59.10%). Another finding in China during 2008–2011 showed that *P. aeruginosa* was the predominant Gram-negative bacterium in the general ward (20.50%), while *A. baumannii* was the predominant Gram-negative bacterium in the RICU (33.88%) (He et al., 2014). In the two wards of our hospital, *P. aeruginosa*, *A. baumannii*, and *K. pneumoniae* had rather high infection prevalence, and their combined infection rate was even over 70% in the RICU. Consistent with our research, studies in United States during early years found *P. aeruginosa* (17.0%) was relatively common organism isolated in ICU with respiratory infections (Fridkin, 2001), and recently several reports from Asian countries such as Indonesia, Thailand revealed that *P. aeruginosa* (26–50%) was the most frequent bacterium in ICU followed by *K. pneumoniae* (15%) as well (Refdanita et al., 2010; Radji et al., 2011).

The distribution of bacterial strains isolated from LRTI patients’ blood cultures in the present study demonstrated the proportion of Gram-positive bacteria in the pediatric ward (88.80%) and adult respiratory ward (61.70%) were significantly higher than Gram-negative bacterial strains. The dominant pathogens of two wards both belonged to *Staphylococcus* sp., as *S. aureus* attributed to 21.8% in the adult and *S. epidermidis* attributed to 47.20% in children, indicating that it is of vital importance to reduce staphylococcal infections in our hospital. Armanian et al. (2019) reported a similar finding that *S. epidermidis* (53.1%) was the most common species in blood cultures of infants with infections in the neonatal internal wards and neonatal intensive care unit (NICU). Some researchers observed that the most common organism identified in blood cultures from pediatric LRTIs was *S. pneumoniae* 77% (Iroh Tam et al., 2015) to 78.5% (Neuman et al., 2017), 92% of which were susceptible to penicillin (Neuman et al., 2017). Another report showed that two of 177 blood cultures finally reported as true positive were *S. pneumoniae* (Davis et al., 2017). However, among the 125 positive cultures in our pediatric ward, only two were *S. pneumoniae*. The possible explanation could be due to the facts that LRTI patients might have taken antibiotics at home or in the clinic before admission to hospital and their conditions did not progress to a large number of positive blood cultures with *S. pneumoniae* isolated as it is very sensitive to penicillin (Neuman et al., 2017). Gram-negative bacteria accounting for more than 48% remained the major microorganisms involved in LRTIs from the RICU. Its dominant pathogen was *K. pneumoniae* (27.57%), followed by *S. hominis* (8.65%) and *A. baumannii* (7.57%). Similar to previous reports in China (Tao et al., 2011) and European (Zarb et al., 2012; Wałaszek et al., 2018), the common distribution of RICU infections were lower respiratory tract and bloodstream with the several frequently isolated pathogens, such as *A. baumannii*, *K. pneumoniae*, and *Staphylococcus* sp. Lee et al. (2017) demonstrated that increasing resistance among bloodstream isolations with increasing age, and a probably consequent reduction in the percentage of appropriate empirical antibiotic therapy in elderly.

Inappropriate application of antibiotics will result in antimicrobial resistance further increasing the health-care cost and mortality. Antibiotic resistance is a rapidly increasing global emergency that calls for action from all of society. The resistance patterns such as CRAB and CRE [including carbapenem-resistant *P. aeruginosa* (CRPA), carbapenem-resistant *K. pneumoniae* (CRKP), etc.] have been increasing in recent years. In order to achieve appropriate therapeutics, updated epidemiology of antimicrobial sensitivity is required to support therapeutic guidelines. A Chinese multicenter epidemiology survey of pneumonia revealed that the detection rates of CRAB and CRPA were 78.9 and 70.7% (Zhao et al., 2013). In the present study, we observed *P. aeruginosa* of LRTI patients remained highly susceptible (>70%) to routine antibiotics in pediatric ward, but only had high susceptibility to amikacin, tobramycin, gentamicin in both the adult respiratory ward and RICU. Moreover, its antibiotic sensitivity to meropenem and imipenem was moderate in the adult respiratory ward, but mild (<30%) in the RICU. *A. baumannii* remained highly or moderately susceptible to tested antibiotics in the pediatric ward, whereas it exhibited relatively low drug-susceptibility to antibiotics except tigecycline in the respiratory ward and RICU. *A. baumannii* is highly resistant to antimicrobials especially in ICU and older patients (Goel et al., 2009; Ghanshani et al., 2015), and antibiotic resistance also increased significantly compared to previous decade in LRTIs (Kumari et al., 2007; Goel et al., 2009). *K. pneumoniae* was highly susceptible to tigecycline of patients hospitalized with LRTIs in all three wards. Its drug-susceptibility to piperacillin/tazobactam, imipenem, meropenem, amikacin, tobramycin, and trimethoprim/sulfamethoxaz was high in both the pediatric ward and adult respiratory ward, but mildly susceptible to routine antibiotics in RICU. A current research in China showed that the detection rates of CRKP, CRPA, and CRAB in tertiary hospitals were significantly higher than in secondary hospitals (Tang et al., 2018). The phenomenon may result from inadequate empirical use of antimicrobials in primary hospitals or secondary hospitals, and the multidrug-resistant bacteria emerged before the submission into tertiary hospitals which then prescribed more broad-spectrum antibiotics in tertiary hospital. As a predominant Gram-positive bacterium, *S. aureus* isolated from LRTI patients was highly susceptible to several antibiotics. However, its sensitivity to benzylpenicillin, oxacillin, levofloxacin, moxifloxacin, and erythromycin was relatively low in the RICU. It is revealed in a multicenter survey on HAP that the rate of methicillin-resistant *S. aureus* (MRSA) is 87.8% (Zhao et al., 2013). As MRSA exhibits high rates of morbidity and mortality and can cause metastatic or complicated infections (Hassoun et al., 2017), it remains a major healthcare issue. *S. pneumoniae* is reported very sensitive to penicillin (Neuman et al., 2017). A similar finding in our study showed that *S. pneumonia* was the second most common bacterium in the pediatric ward, and its sensitivity to the antibiotics except trimethoprim/sulfamethoxazole was high in the pediatric ward and adult respiratory ward.

The rate of Gram-negative infections including *Pseudomonas* sp., *Acinetobacter* sp., and *Klebsiella* sp., differs across various countries or regions with highest rate in Asia, and least in North America and Western Europe (Vincent et al., 2009). High prevalence of resistant species in developing countries could be due to non-compliance in infection control regulations and to lack of or non-compliance with antibiotic policy. Lee et al. (2017) demonstrated that significant positive age-related trends were noted in antibiotic-resistant pathogens, and the decreased percentage of appropriate antimicrobial therapy in patients was associated with increased mortality, which suggested different antibiotic therapies should be considered for distinct age groups.

However, our research has certain limitations, partly because it was only focused on LRTI patients from one tertiary hospital in Beijing, which might have led to enrollment bias considering the geographic location. Viruses have been identified in many pneumonia episodes; however, ascribing pathogenicity may be difficult unless they are invariably associated with disease (Marangu and Zar, 2019). These include respiratory syncytial virus, rhinovirus influenza, parainfluenza, human metapneumovirus, adenovirus, and parainfluenza (Shi et al., 2015; Marangu and Zar, 2019). In this report, we only focused on bacterial distribution and antibiotic susceptibility of LRTI patients, which is one of limitations. Our study is also limited by preliminary and retrospective cross-sectional design. Therefore, a multicenter, longitudinal, prospective research is required to confirm our findings.

## Conclusion

The distribution of prevalent microorganisms and their patterns of antibiotic susceptibility revealed a high divergence among LRTI patients admitted to the pediatric ward, adult respiratory ward, and RICU in our hospital. Thus, different antibiotic therapies for distinct age groups were suggested, and overuse of broad-spectrum antibiotics should be avoided.

## Data Availability Statement

All datasets generated for this study are included in the article/supplementary material.

## Ethics Statement

The studies involving human participants were reviewed and approved by Institutional Ethics Committee of Peking University First Hospital. Written informed consent for participation was not required for this study in accordance with the national legislation and the institutional requirements.

## Author Contributions

ND and JD contributed to conceptualization, methodology, formal analysis, investigation, and writing the original draft. CH contributed to validation and reviewing and editing the manuscript. HL was involved in conceptualization, reviewing and editing the manuscript, and supervision. All authors contributed to the article and approved the submitted version.

## Conflict of Interest

The authors declare that the research was conducted in the absence of any commercial or financial relationships that could be construed as a potential conflict of interest.
